# Templated Synthesis of Exfoliated Porous Carbon with Dominant Graphitic Nitrogen

**DOI:** 10.1021/acsmaterialsau.2c00074

**Published:** 2023-02-27

**Authors:** Esmail Doustkhah, Ahmed Kotb, Saeede Tafazoli, Timuçin Balkan, Sarp Kaya, Dorian A. H. Hanaor, M. Hussein N. Assadi

**Affiliations:** † Koç University Tüpraş Energy Center (KUTEM), 34450 Sarıyer, Istanbul, Turkey; ‡ Chemistry Department, Faculty of Science, Al-Azhar University, 71524 Assiut, Egypt; § Materials Science and Engineering, Koç University, 34450 Sarıyer, Istanbul, Turkey; ∥ n2STAR Koç University Nanofabrication and Nanocharacterization Center for Scientific and Technological Advanced Research, 34450 Sarıyer, Istanbul, Turkey; ⊥ Department of Chemistry, Koç University, 34450 Sarıyer, Istanbul, Turkey; # Fachgebiet Keramische Werkstoffe, Technische Universität Berlin, 10623 Berlin, Germany; △ RIKEN Center for Emergent Matter Science, 2−1 Hirosawa, Wako, Saitama 351-0198, Japan

**Keywords:** Hard template, Graphitic nitrogen, Carbon nitride, Supercapacitance, 2D hard template, Graphitic carbon nitride, g-C_3_N_4_

## Abstract

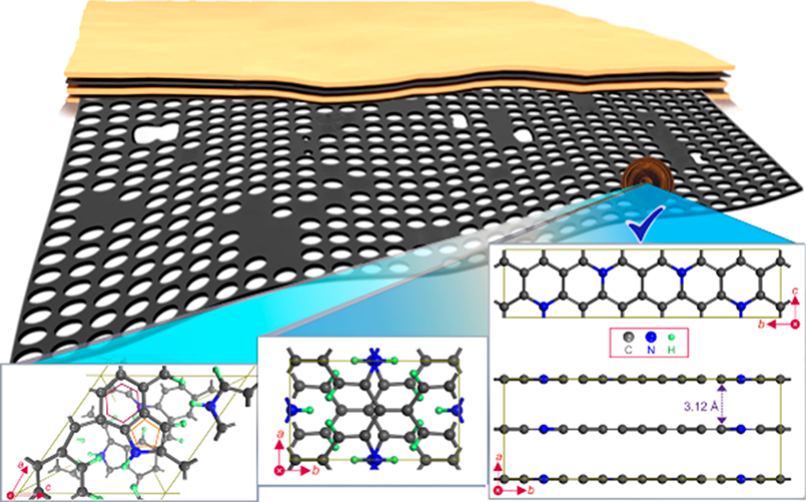

We present here a new approach for the synthesis of nitrogen-doped porous graphitic carbon (g-NC) with a stoichiometry of C_6.3_H_3.6_N_1.0_O_1.2_, using layered silicate as a hard sacrificial template. Autogenous exfoliation is achieved due to the heterostacking of 2D silicate and nitrogen-doped carbon layers. Micro- and meso-porosity is induced by melamine and cetyltrimethylammonium (C_16_TMA). Our density functional calculations and X-ray photoelectron spectroscopy (XPS) observations confirm that the most dominant nitrogen configuration in g-CN is graphitic, while pyridinic and pyrrolic nitrogens are thermodynamically less favored. Our large-scale lattice dynamics calculations show that surface termination with H and OH groups at pores accounts for the observed H and O in the composition of the synthesized g-NC. We further evaluate the electrocatalytic and the supercapacitance activities of g-NC. Interestingly, this material exhibits a specific capacitance of ca. 202 F g^–1^ at 1 A g^–1^, retaining 90% of its initial capacitance after 10,000 cycles.

## 1 Introduction

The distinct physical properties of graphitic layered carbons, coupled with their chemical stability and relatively low costs, make them suitable toward applications such as electrocatalysis,^1−5^ and energy conversion/storage.^6,7^ Graphitic materials in general, with or without nitrogen substitution, are initially stacked and therefore exhibit comparatively low surface reactivity and mass diffusion.^8,9^ These shortfalls motivate research toward the efficient production of functionalized layered structures with high levels of reactive surfaces.^10−13^ Specifically, those techniques that can autogenously produce porous, exfoliated, and functionalized layered carbon structures are sought after. Such techniques can also eliminate the cumbersome multistep postmodification procedures. In this regard, montmorillonite and MXenes as 2D materials have appeared as hard templates for obtaining self-exfoliated porous carbon structures that exhibit excellent electrochemical activity.^14−17^ Carbon nanostructures from such approaches also show superior electric double-layer capacitor activities than traditionally prepared graphene-based materials.^14−17^

Nitrogen-substituted and exfoliated graphitic carbon materials have significant potential to improve functional properties in various applications.^12,18^ This material class is generally referred to as graphitic N-doped carbon (g-NC). Here, nitrogen substitution in graphene layers occurs in three forms: pyridinic, pyrrolic, and graphitic configurations, each denoting a distinct placement of nitrogen in the lattice.^19^ Pyridinic nitrogen substitution in graphitic carbon occurs at the boundaries of the 2D graphene lattice or defect sites such as vacancies in graphene planes. Such pyridinic nitrogen atoms bond, relatively weakly, to two carbons while sharing a π electron in graphene’s delocalized π bonds. Dangling bonds in this form of nitrogen make this species reactive and less stable. Pyrrolic nitrogen substitution occurs at 5-membered rings in graphitic carbon, involving the contribution of two π-electrons to the π bonding. Graphitic nitrogen substitutes for carbon in the graphene structure and bonds to three carbon atoms. The higher thermal and chemical stability of graphitic nitrogen species make these desirable for diverse applications such as sustainable electrocatalysis^20^ or metal supporting for (electro)catalysis.^21^ Although graphitic nitrogen is less reactive than pyridinic nitrogen, in some cases, graphitic nitrogen exhibits a superior electrocatalytic activity than pyridinic nitrogen and the mixture of pyridinic and graphitic.^20^ Since most synthetic methods cause a mixture of pyridinic/graphitic nitrogen substitution,^22^ developing synthetic methods toward more dominant graphitic nitrogen substitutions is still necessary.

The distinction between forms of nitrogen speciation in carbon can be readily achieved based on their bonding energy via X-ray photoelectron spectroscopy (XPS) analysis and is of considerable importance in organic chemistry and materials design. The speciation of N substitution in a graphene layer depends on the carbon-to-nitrogen ratio and the synthesis protocol of the material with implications on the electronic and magnetic properties and, by extension, the material’s functional properties. Further understanding of nitrogen speciation in synthetic carbon structures is required to allow for effective materials design.^12^ In the present work, we have utilized a layered sodium silicate as a 2D hard template for the intercalation of melamine and the production of exfoliated graphitic porous carbon with a carbon-to-nitrogen ratio of ∼6:1. In this regard, cetyltrimethylammonium (C_16_TMA^+^) contributes to the intercalation of melamine by serving as a preintercalating agent in the interlayers of silicates through exchanging with Na^+^ of the sodium silicate. As we show here, the synthesized g-NC exhibits promising supercapacitance and electrocatalytic activity toward the oxygen reduction reaction (ORR). We also, through theoretical simulations, predict the g-NC’s crystal structure with graphitic, pyridinic and pyrrolic nitrogen at this N content. By benchmarking our spectroscopic measurements against the simulated structures, we demonstrate that graphitic nitrogen exhibits greater stability and electrical conductivity than the other two nitrogen configurations.

## 2 Materials and Methods

### 2.1 Synthesis of Graphitic N-Doped Carbon

In the first step, sodium silicate (purchased from Nippon Chemical Industrial Co. Ltd.) was dispersed in the aqueous solution of C_16_TMACl (0.1 M, FUJIFILM Wako Pure Chemical Corporation, 95%) and stirred for 2 days at room temperature, according to a reported method.^23^ Then, a precipitate was collected by centrifugation and washed with distilled water four times. Then, the obtained powder was dispersed in 2 wt % melamine/DMSO solution, stirred for 1 day, separated by centrifugation, washed with EtOH, and dried at room temperature in a vacuum for 10 h. Then the obtained material (C_16_TMA-SiO_2_-mel) was pyrolyzed at 800 °C with a heating rate of 1 °C/min and a dwell time of 5h in N_2_. Then, the obtained powder was dispersed in HF solution (10 wt %) to remove the silica template. Eventually, the black powder (g-NC) obtained by centrifugation was washed with distilled water and freeze-dried. The CHNS analysis of g-NC (Table S1) showed that the final formula of the synthesized g-NC is C_6.3_H_3.6_N_1_O_1.2_.

### 2.2 Characterization

XRD patterns of the powder samples were collected using a powder X-ray diffractometer (Smart Lab, RIGAKU) with Cu Kα radiation at 40 kV and 30 mA. A HITACHI SU-8000 was used to measure the scanning electron microscope (SEM) images to monitor the morphologies of all samples. Transmission electron microscopy (TEM) images were recorded on a JEOL JEM-2100F microscope (operated at 300 kV). Atomic force microscopy (AFM) images were recorded on a SPA-400 system (SPA400, Seiko Instruments Inc.). N_2_ adsorption–desorption measurement was performed with a BELSORP-mini Autosorb (Japan, the samples were evacuated at 120 °C for 6 h) at 77 K in liquid N_2_ temperature. FTIR measurement was carried out with a Shimadzu FTIR-4200. *In situ* and *ex situ* Raman spectra were performed using a Renishaw inVia spectrometer. The setup of *in situ* Raman spectroscopy is presented in Figure S1. The thermal gravimetry-differential thermal (TG-DT) analyses were carried out on a Pt cup in N_2_ atmosphere via a Rigaku Thermo plus TG8120 apparatus. The cyclic voltammetry (CV) and linear sweep voltammetry (LSV) tests were performed at 10 mV s^–1^.

### 2.3 Computational Settings

Spin polarized density functional calculations were performed using the VASP code,^24^ with projector-augmented wave potentials^25^ and Perdew–Burke–Ernzerhof functional.^26,27^ The Brillouin zone was sampled with a very tight *k*-point mesh with a maximum of 0.018 Å^–1^ spacing, generated using the Monkhorst–Pack scheme.^28^ All other electronic parameters were set using the precision key “*accurate*”, ensuring sufficient numerical convergence regarding the cutoff energy for the plane-wave-basis and the fast Fourier transformation mesh. van der Waals dispersion energy correction was applied based on the Grimme *et al.* method.^29^ The adequacy of these settings for carbon-based materials was established in our previous studies.^30,31^ For geometry optimization, all atomic coordinates and lattice parameters were allowed to relax to force components smaller than 0.001 eV/Å with an energy threshold of 10^–7^ eV/atom. The optimized structures were examined for symmetry using the symmetry detection script FINDSYM.^32^ Classical lattice geometry optimization for large nanoparticles was performed using the GULP code^33,34^ and ReaxFF force field.^35,36^ Here, energy and force thresholds were set at 10^–4^ eV/cell and 10^–2^ eV/Å per component. This force-field-based geometry optimization was achieved within ∼22,000 cycles.

### 2.4 Supercapacitance Measurements

For supercapacitance tests, a homogeneous ink was prepared as follows: 4.0 mg of the candidate supercapacitor powder was dispersed in isopropanol:water (380 μL, 2:1) containing Nafion (5.0 wt %, 20 μL). After 30 min of sonication, 100 μL of the suspension was dropped onto a carbon paper (thickness: 1 mm) with an area of 1 × 1 cm^2^ and dried overnight at 60 °C. The mass loading was 1 mg cm^–2^. All electrochemical measurements were carried out using a CHI 660E instrument, and the CV and galvanostatic charge–discharge (GCD) measurements were carried out in 3 M KOH between −0.8 and 0.0 V vs SCE, with a platinum wire and saturated calomel electrode (SCE) as a counter and a reference electrode, respectively. The long-term stability test for g-NC was conducted by cycling between −0.8 and 0.0 V vs SCE in 3 M KOH at a scan rate of 100 mV s^–1^.

The gravimetric specific capacitances (*C*_g_, F g^–1^) of the as-made electrodes were calculated from the GCD curves using the following equation:
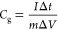
1Here, *I* is the discharge current (A), Δ*t* is the discharge time difference (s), *m* is the mass of the active material (g), and Δ*V* is the potential change during the discharge process (V).

## 3 Results and Discussion

### 3.1 Characterizing Porous g-NC

**Scheme 1:**
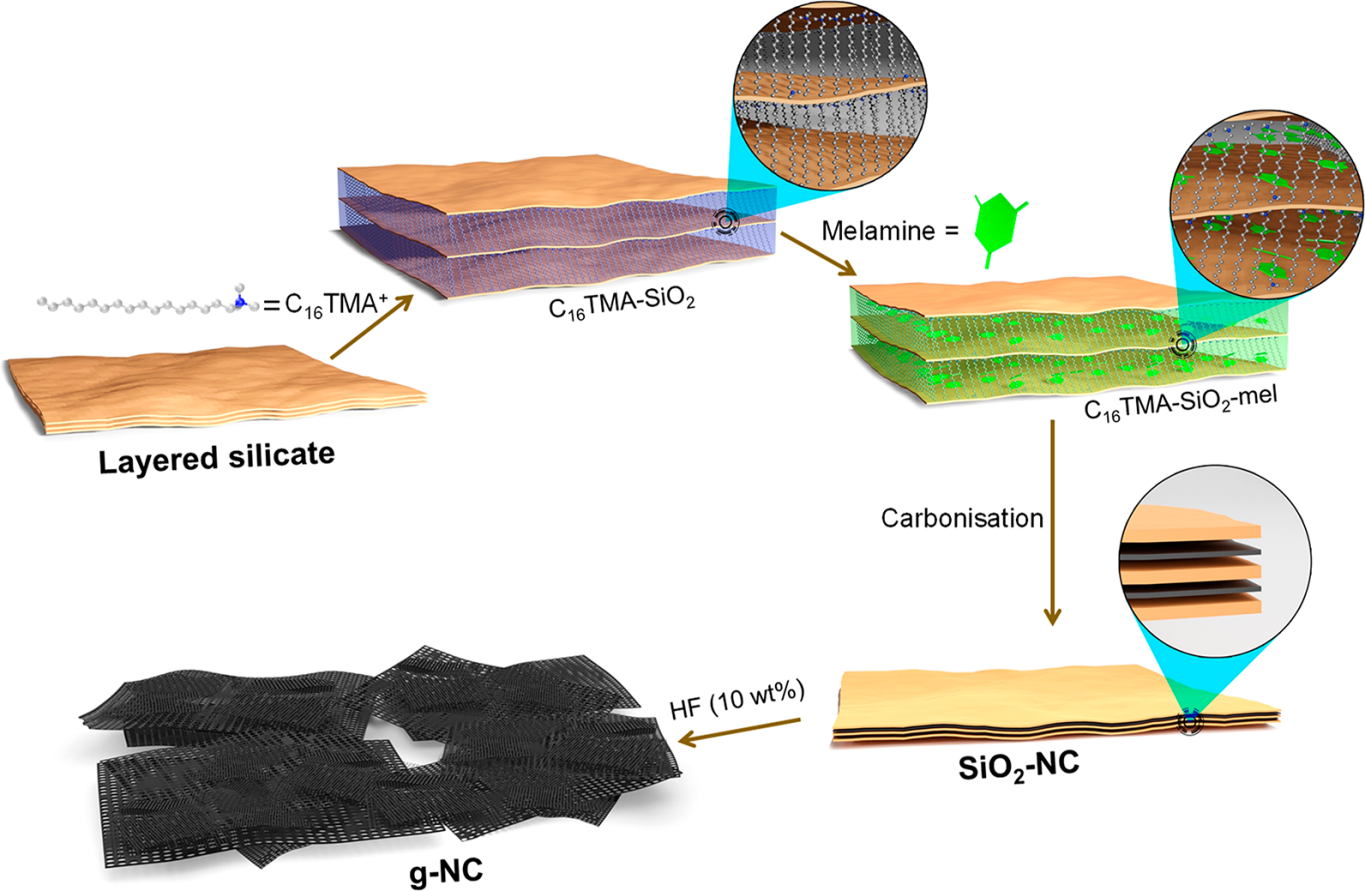
Synthetic Scheme of g-NC through Hard-Templating

We used a hard templating approach to obtain N-doped layered porous carbon (g-NC) samples. Sodium silicate was used as a 2D hard template to induce a 2D layered structure to the final carbonized structure. Then, cetyltrimethylammonium (C_16_TMA^+^) was utilized to expand the interlayers’ space in layered silicate and make the interlayers accessible for the intercalation of g-NC precursor (i.e., melamine). In addition to preintercalation role, C_16_TMA^+^, as an amphiphilic alkylammonium compound,^37^ favors the intercalation of both hydrophilic- and hydrophobic precursors. Thus, the intercalation of melamine as carbon and nitrogen precursor (C_3_N_6_H_6_) can be further facilitated by C_16_TMA^+^ present in the interlayers of C_16_TMA-SiO_2_. Once the melamine was successfully intercalated (C_16_TMA-SiO_2_-mel), we pyrolyzed the sample at 800 °C in N_2_ atmosphere, which led to the production of layer-by-layer carbon assembly and layered silicate, which we refer to as SiO_2_–NC. The pyrolysis of C_16_TMA-SiO_2_-mel, as studied by TG-DT analysis, involves a mass loss of 35%, predominantly in the range of 200–600 °C (Figure S2). Note that the preintercalated C_16_TMA^+^ can copyrolyse with melamine during carbonization, thus, a fraction of the g-NC originates from the pyrolysis of C_16_TMA^+^.^23^ After achieving this heteroassembly of the layered carbon and silicate structure, we etched the silicate to produce a new holey graphitic nitrogen-doped carbon in exfoliated form, which we refer to as g-NC. This autogenous exfoliation originates from the fact that during the etching of silicate layers, g-NC layers are unstacked in the aqueous solution and remain exfoliated until suspended in the methanolic and DMF solutions. Scheme 1 represents the schematic synthesis pathways of the g-NC.

**Figure 1:**
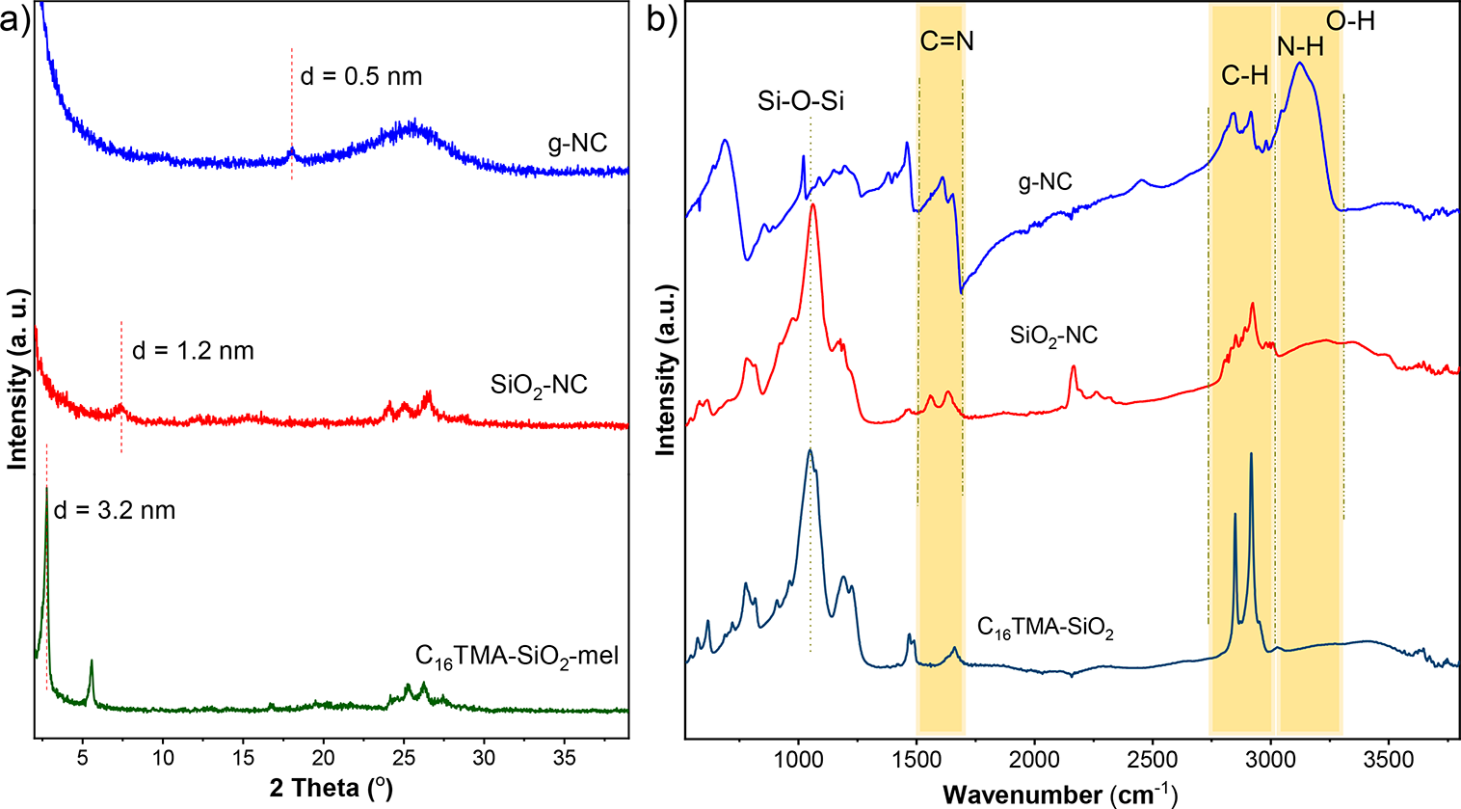
(a) XRD patterns of C_16_TMA-SiO_2_-mel, SiO_2_–NC, and G-NC. (b) ATR-FTIR spectra of C_16_TMA-SiO_2_, SiO_2_–NC, and g-NC (note that since the physical color of g-NC is black, the baseline of the spectra was corrected).

Figure 1a shows the X-ray diffraction pattern of the resultant compound throughout the synthesis procedure. C_16_TMA-SiO_2_-mel showed a significant peak at 2θ = 2.78°, indicative of the C_16_TMA-expanded (001) plane in layered silicate. This *d* value demonstrates that melamine, together with C_16_TMA^+^ occupies ∼2 nm thickness of the interlayers after subtracting the layer’s thickness (∼1.2 nm).^38^ After carbonization, the peak at 2.78° disappeared due to the exfoliation and the shrinkage of the interlayer space. Moreover, after etching SiO_2_–NC, the peaks at 23°–29° disappeared, indicating that the silicate layers were successfully removed. The new broad peak in g-NC at ∼26° corresponds to the (002) plane in g-NC. The appearance of another peak at ∼17° which can be assigned to the (100) plane, likewise the one in graphitic carbon nitrides.^39^

FTIR spectroscopy was used to study the variation of functional groups during each synthetic step (Figures 1b and S3). Starting from lower wavenumber, the presence of a sharp peak in the FTIR spectra of C_16_TMA-SiO_2_ and SiO_2_–NC at ∼1050 cm^–1^ indicates Si–O–Si groups, originating from the silicate structure. The absence of this Si–O–Si peak in the FTIR spectra of g-NC confirms the complete removal of silicate upon etching. Two additional peaks at 1610 and 1636 cm^–1^ indicate the presence of C=N groups in g-NC’s structure. The appearance of some extra peaks above 3000 cm^–1^, besides the aliphatic peaks in 2900–3000 cm^–1^, indicates aromatic C–H groups in the g-NC structure. Moreover, a broad band at 3124 cm^–1^ indicates the presence of N–H and O–H bonds in g-NC, in which the broadness critically depends on the extent of hydrogen bonds.

**Figure 2:**
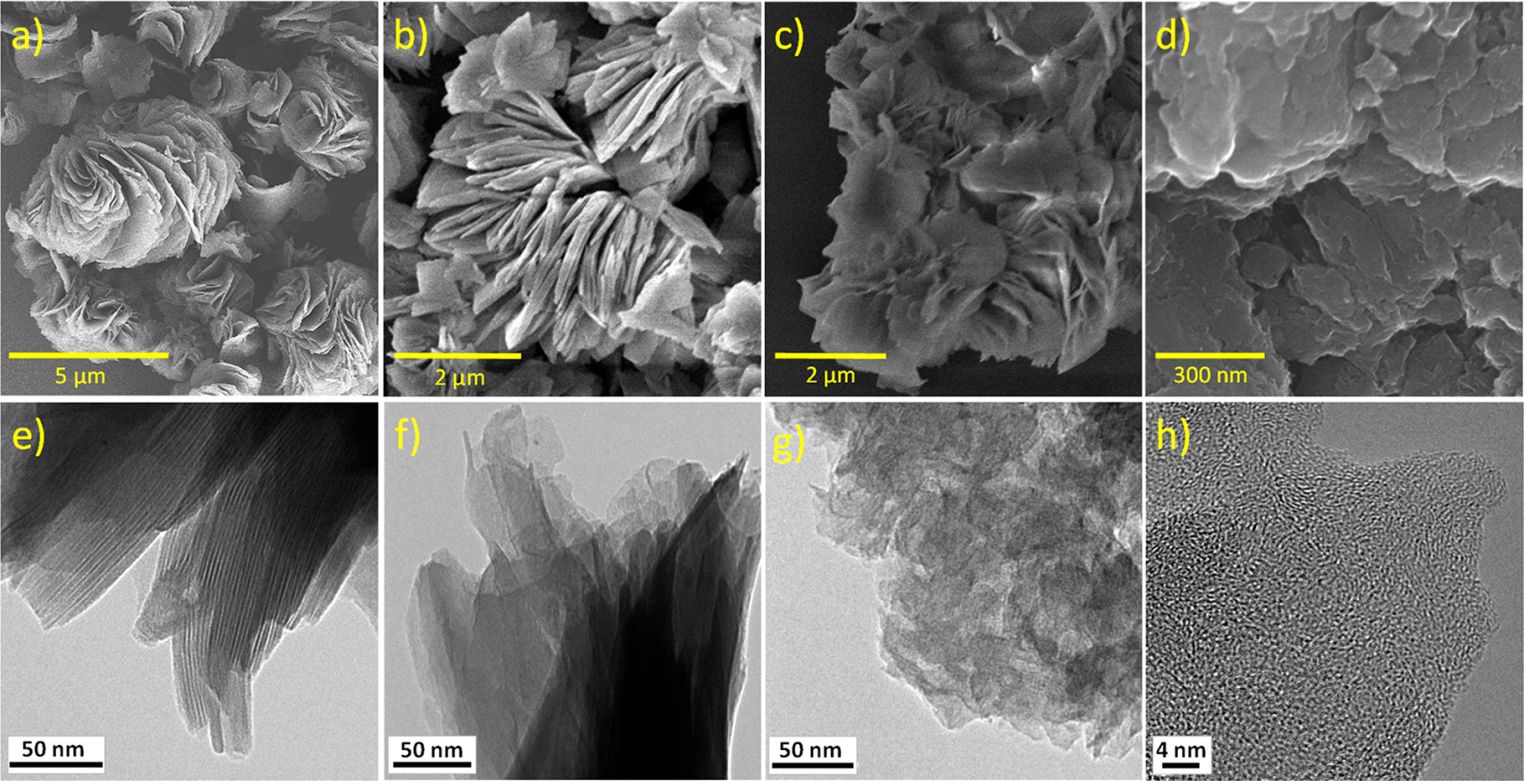
SEM images of (a) layered silicate, (b) C_16_TMA-SiO_2_-mel, (c) SiO_2_–NC, and (d) g-NC. TEM images of (e) C_16_TMA-SiO_2_-mel, (f) SiO_2_–NC, and (g) g-NC. (h) HRTEM image of g-NC.

Comparing the SEM images of C_16_TMA-SiO_2_-mel and sodium layered silicate shows that the structure slightly changes from the stacked pristine form of the layered silicate, confirming that the intercalation slightly disaggregates the particles (Figure 2a and b). However, the microstructure of C_16_TMA-SiO_2_-mel pyrolyzed at 800 °C (Figure 2c) shows loose particles, indicating an effective exfoliation among the stacked layers. The SEM image of g-NC, shows that the average lateral size of the layers is smaller than their template (layered silicate) and appears with a new morphology, mainly exfoliated (Figure 2d). The TEM image of C_16_TMA-SiO_2_-mel also shows that the layers are stacked and in an ordered assembly form. Inspection of the TEM image of SiO_2_–NC also indicates that the thickness of the layers has decreased. A significant observable alteration in the structure and the lateral sizes of the layers after etching g-NC is also discernible by comparing the TEM images of C_16_TMA-SiO_2_-mel and SiO_**2**_-NC with that of g-NC, as presented in Figure 2e–h.

**Figure 3:**
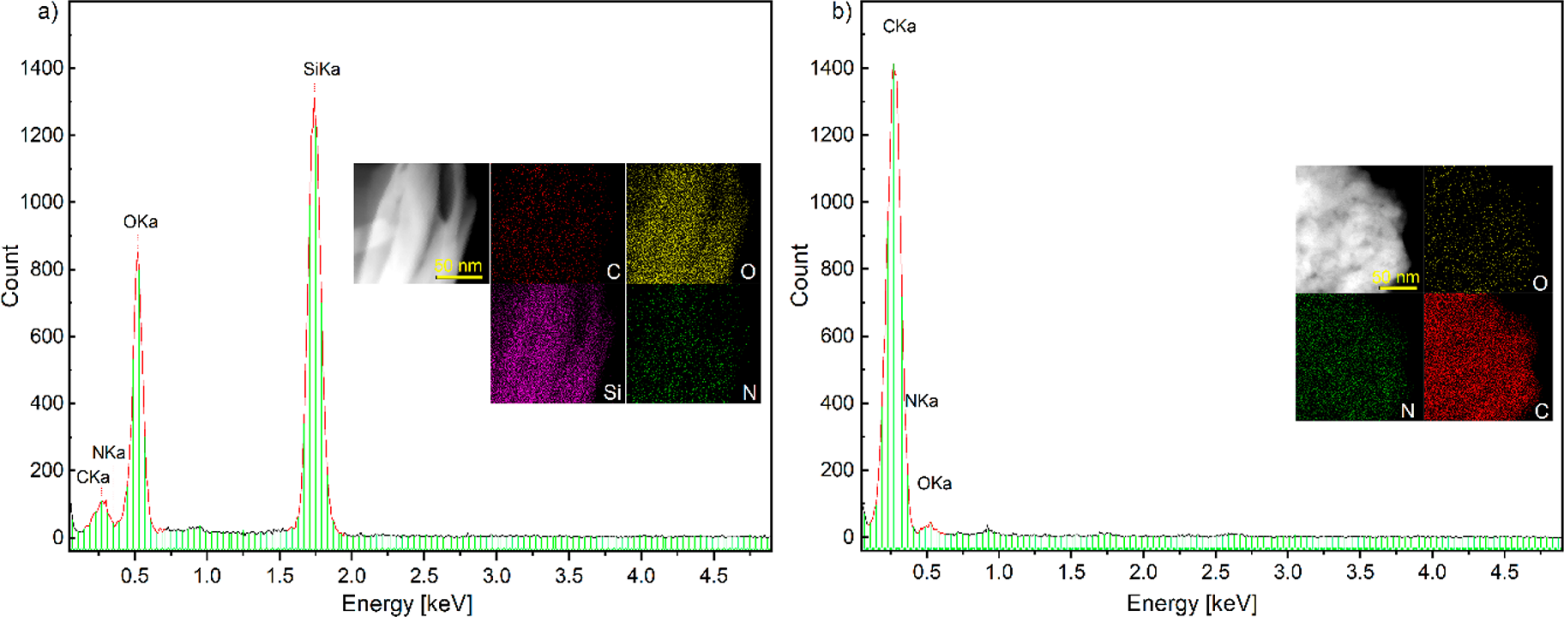
TEM-EDS spectra of (a) NC and (b) g-NC. Inset (a) represents the TEM image and NC and its elemental TEM-mapping images of C, O, Si, and N. Inset (b) represents the TEM image of g-NC and its elemental TEM-mapping abundance of O, N, and C in each corresponding panel.

Elemental TEM mapping images of the elements and TEM-EDS spectra of SiO_2_–NC and g-NC are given in Figure 3a. We conducted the same measurement to pursue the content of the Si and O elements in the final material (g-NC). According to Figure 3b, Si has already been eliminated during the etching, while a small fraction of oxygen has remained within the final material, showing that the O species is covalently bonded to g-NC’s structure. Therefore, these characterizations indicate that the silicate has been fully etched away after treating SiO_2_–NC with HF solution.

**Figure 4:**
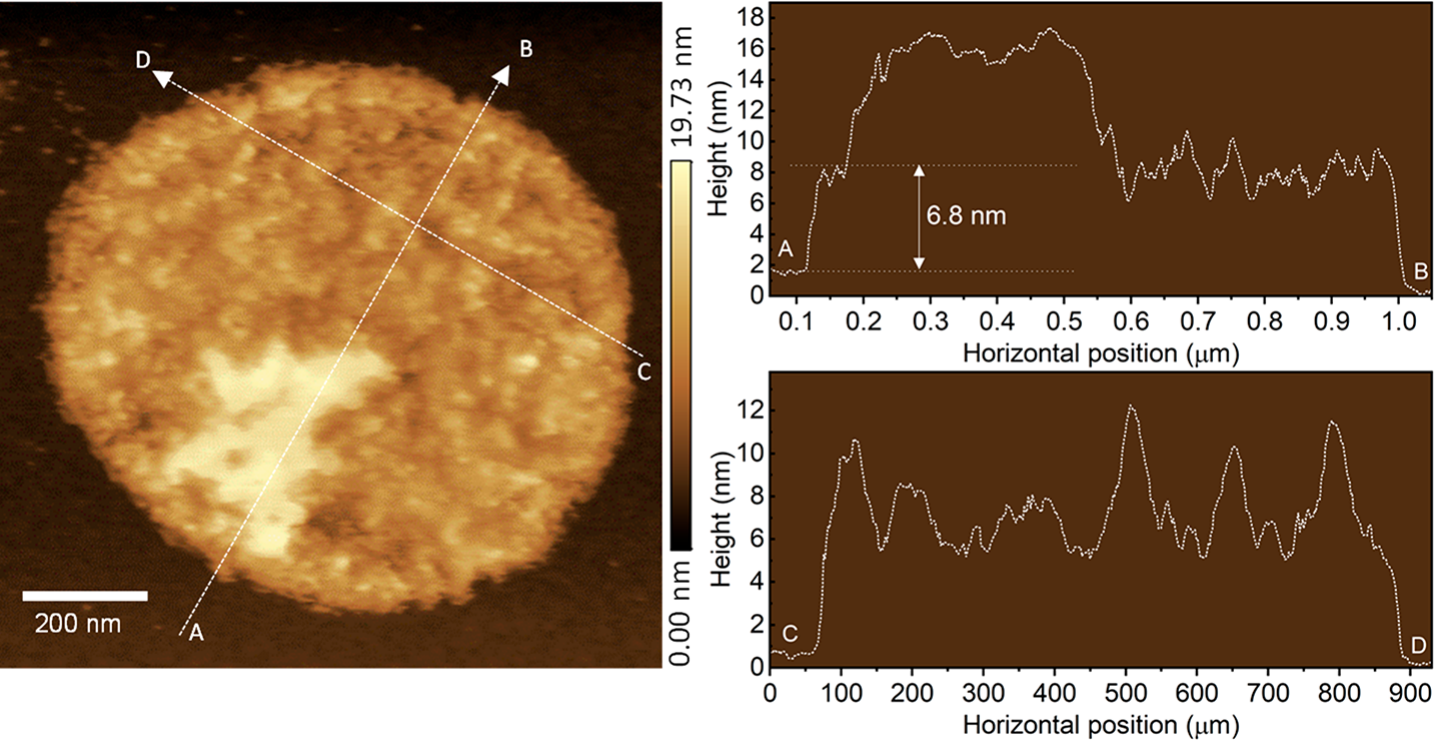
AFM image of SiO_2_–NC partly etched with HF. Right-side panels show the height profiles from two different directions.

Atomic force microscopy was applied here to examine the microporosity and etching process in the layers (Figure 4). Results indicated clear fluctuations in height profiles in the layers’ lateral dimension, showing that the silicate sheets are partially etched, so other than the remaining silicate, the templated g-NC can also be found. Furthermore, the thickness of the captured layer also shows 6.8 nm, which can correspond to solid g-NC exfoliated into a few layers even if there is no remaining silicate among the g-NC layers.

**Figure 5:**
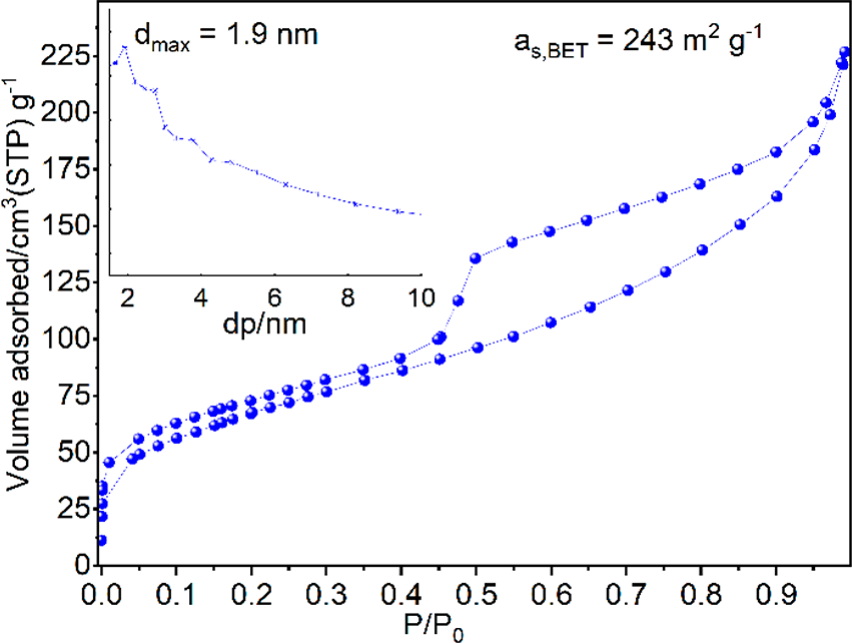
N_2_ adsorption–desorption isotherm of g-NC; inset shows the related BJH plot.

The N_2_ adsorption–desorption isotherm of g-NC, shown in Figure 5, follows a type IV pattern. The hysteresis loop pattern of the physisorption plot also follows the type H4 hysteresis loop pattern, confirming that the micro/mesoporosity is not ordered but rather complex.^40^ The BET plot shows that the specific surface area is 243 m^2^ g^–1^. BJH plot (inset in Figure 5) shows that the pore profile in g-NC is not monosized but rather contains various pore sizes, with pores of ∼1.9 nm being the most common. The pores with a size >2 nm indicate mesoporosity. Note that the disorder mesoporous structure can also originate from interconnected exfoliated layers.^41^

**Figure 6:**
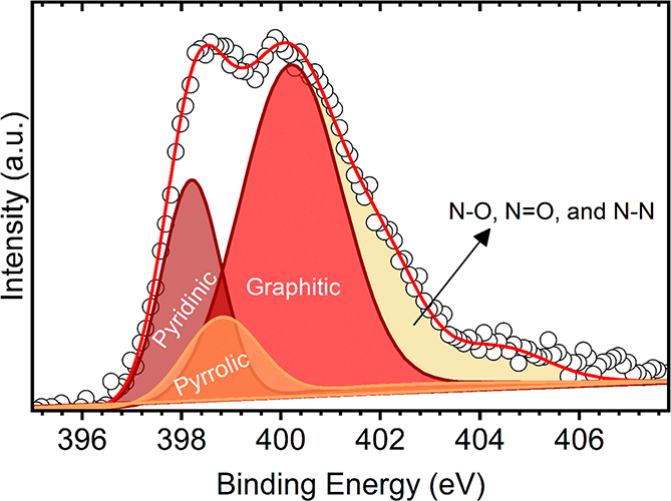
Relevant pyridinic and graphitic nitrogens peaks in XPS spectra of N 1s in terms of binding energy in the synthesized g-NC. For the sake of simplicity, the deconvoluted peaks of other related nitrogen species that have minor quantities are not shown here.

Next, we studied the coordination environment of nitrogen by XPS. Since nitrogen can exist in different configurations, namely graphitic, pyridinic, pyrrolic, and chemisorbed within the carbon structure, identifying the dominant N configurations in g-NC is essential for complete structural characterization. Analyzing the binding energy (BE) positions of N 1*s* orbitals in the XPS spectra yields the ratio of two types of N coordination environments: the doubly bonded 2C–N and the triply bonded 3C-NC. The BE positions of 2C-Ns and 3C-NC are generally variable and depend on features like hydrogen bonds and π-excitations.^42^ However, it is accepted that the doubly coordinated N (2C–N) species, with *sp*^2^ hybridization (e.g., pyridinic and pyrrolic N configurations), possess lower BEs than the triply coordinated N configurations (3C–N), i.e., graphitic and chemisorbed nitrogens.^43^ Details regarding the interpretation of the N’s BE in XPS that we used here can be found in the literature.^43−45^ According to Figure 6, the 3C–N graphitic nitrogen (BE = 400.2 eV) constitutes the major fraction of the nitrogens in the g-NC sample compared to the 2C–N pyridinic (BE = 398.3 eV) and pyrrolic (BE = 398.8 eV). The atomic ratio of graphitic to pyridinic to pyrrolic in g-NC is ∼3:1:0.5, respectively. A small shoulder at BE = 402.1 eV and a bump at BE = 404.3 eV can be ascribed to the minor fraction of the oxidized nitrogen (e.g., NO, NO_2_) and N–N bonded species, respectively (their deconvoluted peak are not provided).^43^

### 3.2 Computational Study

Here, using density functional calculations, we investigate the stability of each N configuration in the g-NC, assuming that each N species possesses a sole configuration. In the context of repeatable lattice structures, there are three distinct configurations where N atoms can be positioned within C’s 2D hexagonal sheets: graphitic, pyridinic, and pyrrolic.^46^ N simply replaces C in the graphitic configuration, bonding to three adjacent C in the 2D hexagonal C sheet. N bonds to two Cs within a hexagonal ring in the pyridinic configuration, implying that pyridinic N is always accompanied by a neighboring C vacancy relative to the intact hexagonal 2D C sheet. In the pyrrolic configuration, N is bonded to two Cs within a pentagonal ring, implying that pyrrolic N is accompanied by two C vacancies: one C vacancy to turn the original hexagonal ring into pentagonal and one to sever a C–N bond. N can also be chemisorbed on the top of the C sheets in a configuration like an adatom.^47^ However, since g-NC was synthesized from melamine containing C and N within its hexagonal rings, the possibility of chemisorbed N configuration is negligible and not investigated here.

We based the search for the g-NC structures, with various N configurations, on building blocks made of the graphite structure. In the absence of nitrogen, our calculations yielded lattice parameters of *a* = 2.465 Å and *c* = 6.961 Å, in excellent agreement with reported experimental values for graphene (*a* = 2.456 Å and *c* = 6.696 Å^48^). We then created supercells of appropriate dimensions and replaced C with N and vacated C sites, when necessary, under the constraint of having stoichiometries close to the experimentally obtained elemental CHNS analysis (Table S1) that predicted an overall C_6.3_H_3.6_N_1_O_1.2_ stoichiometry for g-NC. In other words, we searched for stable structures with either a C to N ratio of 6:1 or 7:1. Furthermore, all dangling bonds created by C vacancies in pyridinic and pyrrolic N configurations were saturated with H. For all reported structures, all distinct possibilities for placing substitutional N or carbon vacancies were investigated, and structures with the lowest total energies were reported.

**Figure 7:**
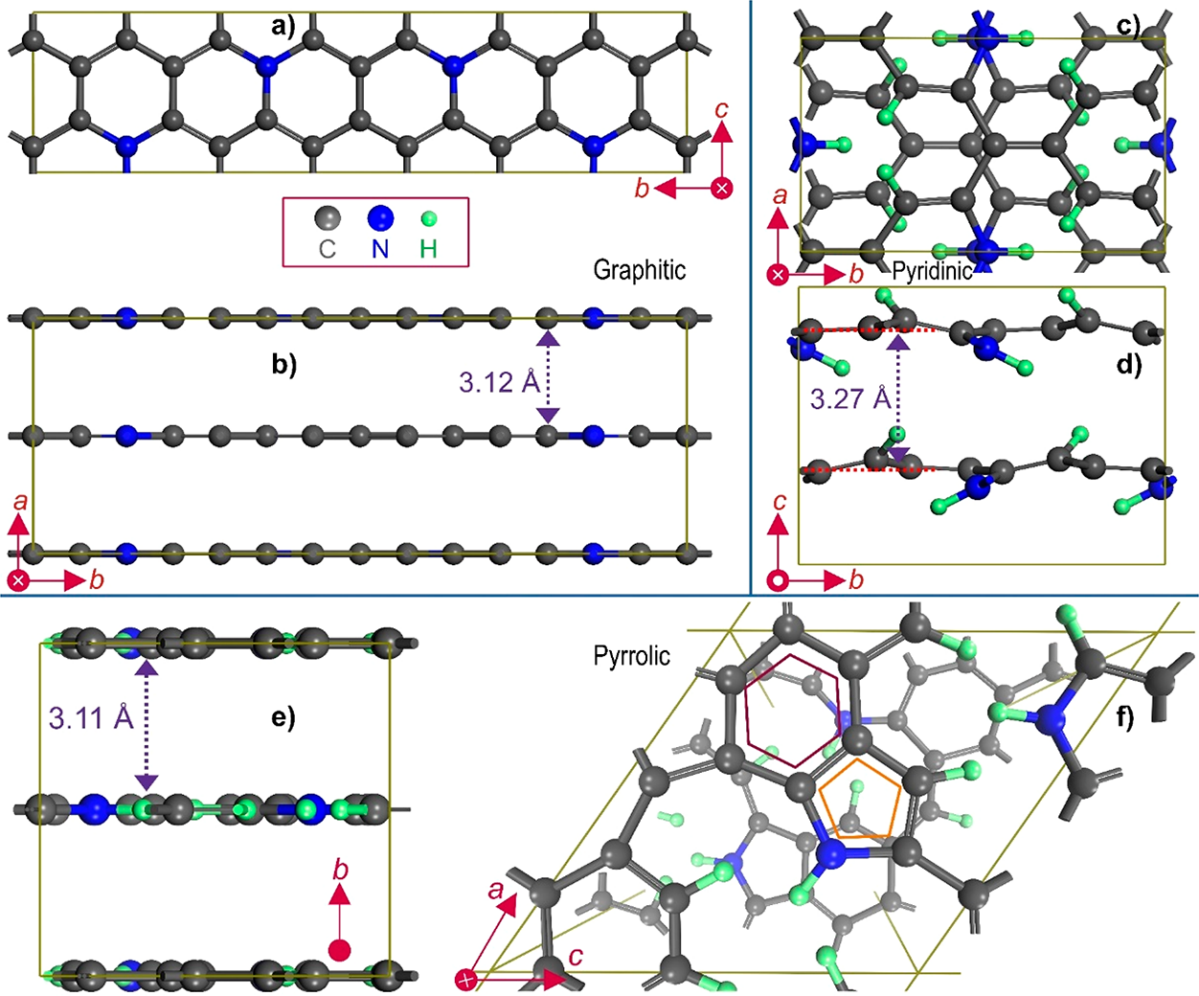
(a,b) Top and side view of the C_6_N_1_ conventional cell with all N at graphitic configuration. (c,d) Top and side view of C_6_N_1_H_3_ conventional cell in which all N are at pyridinic configuration. (e,f) Top and side view of C_7_N_1_H_3_ conventional cell in which all N are at pyrrolic configuration.

For graphitic N, first, we constructed a (√7*a* × √7*a* × 1*c*)*R*19° supercell out of the graphite’s primitive cell, resulting in a supercell containing 28 C atoms in two sheets within the supercell. Four of the C atoms were replaced by N, resulting in a g-NC structure containing a C to N ratio of 6:1, with all N atoms being graphitic. The lattice parameters of the optimized structure are presented in Table 1, labeled as primitive (that is, primitive graphitic C_6_N_1_). A stringent symmetry search, with a tolerance of 10^–5^ Å, on the optimized structure revealed that graphitic g-NC has an orthorhombic symmetry of *Amm*2 (group number 38). As a result, the optimized structure corresponds to a conventional cell for which the lattice parameters are presented in Table 1, and a schematic is shown in Figure 7a-b. For the pyridinic N, we constructed a 2*a* × 2*a* × 1*c* supercell out of the graphite primitive cell, resulting in a supercell containing 16 C atoms in two hexagonal sheets. We then replaced a C with N, vacated a C neighboring the N atom and capped the dangling bonds with H in both hexagonal C sheets in the supercell resulting in a cell with a composition of C_12_N_2_H_6_ (formula unit C_6_N_1_H_3_). The optimized lattice parameters are reported in Table 1, labeled as primitive. Symmetry search detected an orthorhombic symmetry of *Cmc*2_1_ (group no. 36) corresponding to a larger conventional cell for which the lattice parameters are given in Table 1, and the schematics are presented in Figure 7c-d. For pyrrolic N, a stoichiometry of 6 C per 1 N is not attainable with a tractable supercell. Alternatively, as a compromise, we chose to investigate the C to N ratio of 7:1. For this ratio, we constructed a 3*a* × 3*a* × 1*c* supercell out of the graphite primitive cell resulting in a supercell containing 36 C atoms in two hexagonal sheets. In each sheet, we replaced two C atoms with two N atoms in a hexagonal C ring, vacated neighboring C atoms in the N-containing rings, adjusted the bond lengths to create pentagonal rings, and further vacated C atoms from adjacent rings to create the pyrrolic N configuration. After capping the dangling bonds with H, this structure had a composition of C_28_N_4_H_12_ (formula unit C_7_N_1_H_3_). Searching for the most stable placement of these nitrogens and carbon vacancies resulted in the structure reported in Figure 7e-f. Unlike the g-NC containing graphitic or pyridinic N, the predicted g-NC with pyrrolic N had a simpler monoclinic symmetry. All DFT-optimized structures are reported in the CAR format (Data S1–S3), while the symmetrized structures are included in the CIF format in the supporting materials.

**Table 1:** Lattice parameters, composition, and the symmetry of optimized g-NC with the C to N ratio of ∼6:1 reported for both DFT optimized structures, labelled as primitive, and the corresponding symmetrised structures labelled as conventional

	graphitic C_6_N_1_		pyridinic C_6_N_1_H_3_		pyrrolic C_7_N_1_H_3_
space group	*Amm*2 (no. 38)		*Cmc2*_1_ (no. 36)		*P*1*m*1 (no. 6)
structure	primitive	conventional	primitive	conventional	
cell composition	C_24_N_4_	C_48_N_8_	C_12_N_2_H_6_	C_24_N_4_H_12_	C_28_N_4_H_12_
*a* (Å)	8.797	6.240	4.995	5.075	7.946
*b* (Å)	8.797	17.079	4.9945	8.604	6.219
*c* (Å)	6.240	4.227	6.530	6.530	8.136
α (deg)	90.00	90.00	90.00	90.00	90.00
β (deg)	90.00	90.00	90.00	90.00	124.78
γ (deg)	152.20	90.00	61.07	90.00	90.00

**Figure 8:**
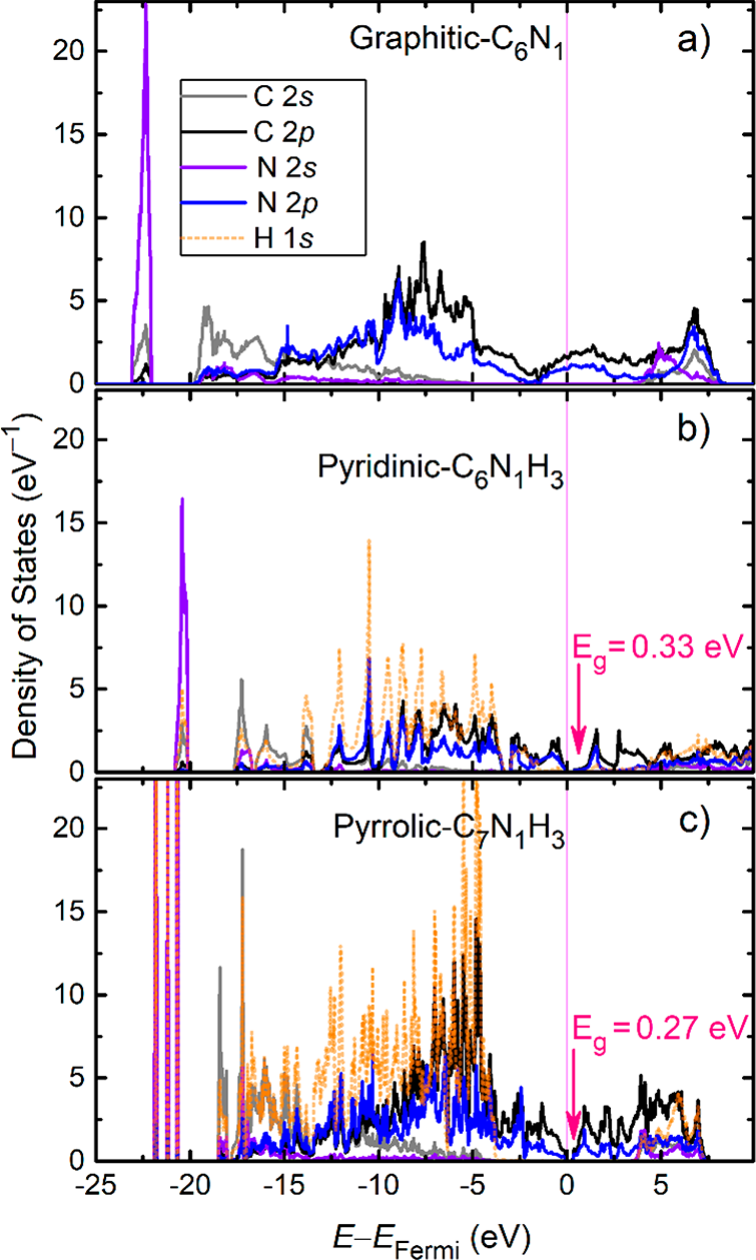
Partial density of g-NC with (a) graphitic, (b) pyridinic, and (c) pyrrolic N configurations.

Next, we examine the relative stability of the identified g-NC structures. To do so, we calculated their formation enthalpy (Δ*H*) relative to graphite according to the following equation:
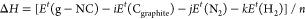
2*E*^*t*^ is the DFT total energy, calculated for the g-NC configurations, graphite, gaseous nitrogen, and gaseous hydrogen. The factors *i*, *j*, and *k* are to be adjusted for balancing the chemical equation, and *n* is the number of atoms in the g-NC structure. Δ*H* was calculated to be 0.100 eV/atom for graphitic g-NC, 0.199 eV/atom for pyridinic g-NC, and 0.291 eV/atom for pyrrolic g-NC. These Δ*H* values indicate that the graphitic g-NC is the most stable among the investigated structures, as it has the lowest Δ*H*. For pyridinic and pyrrolic g-NC, Δ*H* is about twice and three times larger than the graphitic configuration, respectively, indicating a lower probability of formation, corroborating the XPS results given in Figure 6. Furthermore, the positive Δ*H* values demonstrate that the g-NC structures with a C to N ratio of ∼6:1 are merely metastable relative to graphite, further signifying the role of hard-templating in stabilizing these compounds.^49,50^ Additionally, the density of states in Figure 8 shows that, among these three configurations, only graphitic g-NC is readily conductive, similar to graphitic carbon nitride (g-C_3_N_4_).^51,52^ However, the other two configurations have narrow band gaps and are insulating.

**Figure 9:**
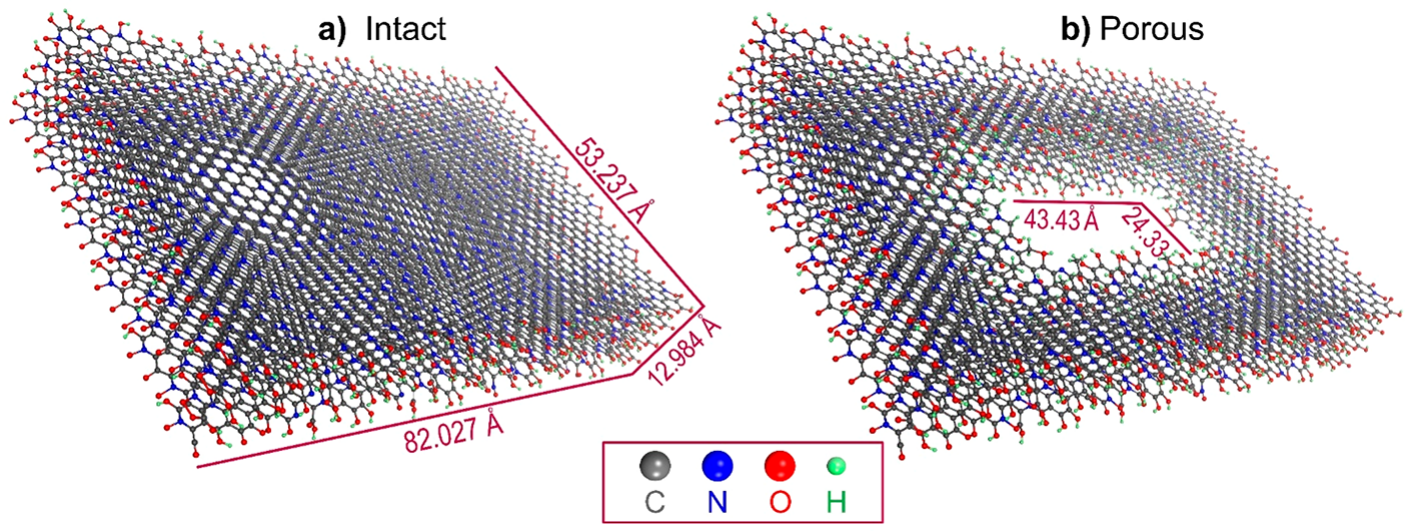
(a) Flat and nonporous nanostructure graphitic C_6_N_1_ capped at the edges with O or OH. (b) Holey flat nanostructure made of graphitic C_6_N_1_.

Lastly, we performed classical lattice dynamics for a large nanoparticle of graphitic g-NC (the most stable configuration) to investigate how pores affect the chemical composition and surface area. Figure 9a shows an intact cuboid graphitic g-NC with edges capped with O or OH, depending on the charge at the dangling bonds created by cleavage. This structure is made of 6664 atoms, has a composition of C_5108_N_872_O_467_H_217_ (nominally C_6_N_1.04_O_0.56_H_0.26_), a mass of 81256.6 amu, and a surface area of 122.46 nm^2^. Figure 9b shows a nanoparticle similar in dimensions to the one in Figure 9a, except with a pore in the middle. This structure is made of 5506 atoms, has a composition of C_3688_N_636_O_625_H_557_ (nominally C_6_N_1.03_O_1.02_H_0.91_), a mass of 63765.8 amu, and a surface area of 161.19 nm^2^. Here, we see that by creating a pore equivalent of removing ∼21% of the nanoparticle’s mass, the surface area increases by ∼31%. Furthermore, since the dangling bonds at the added surfaces were capped with O or OH groups, the holey nanostructure has twice as many O atoms and three times as H atoms compared to the intact structure, explaining the observed H and O concentrations in the synthesized materials even though the graphitic g-NC unit cell does not contain H or O in its bulk form.

### 3.3 Performance as a Supercapacitor

**Figure 10:**
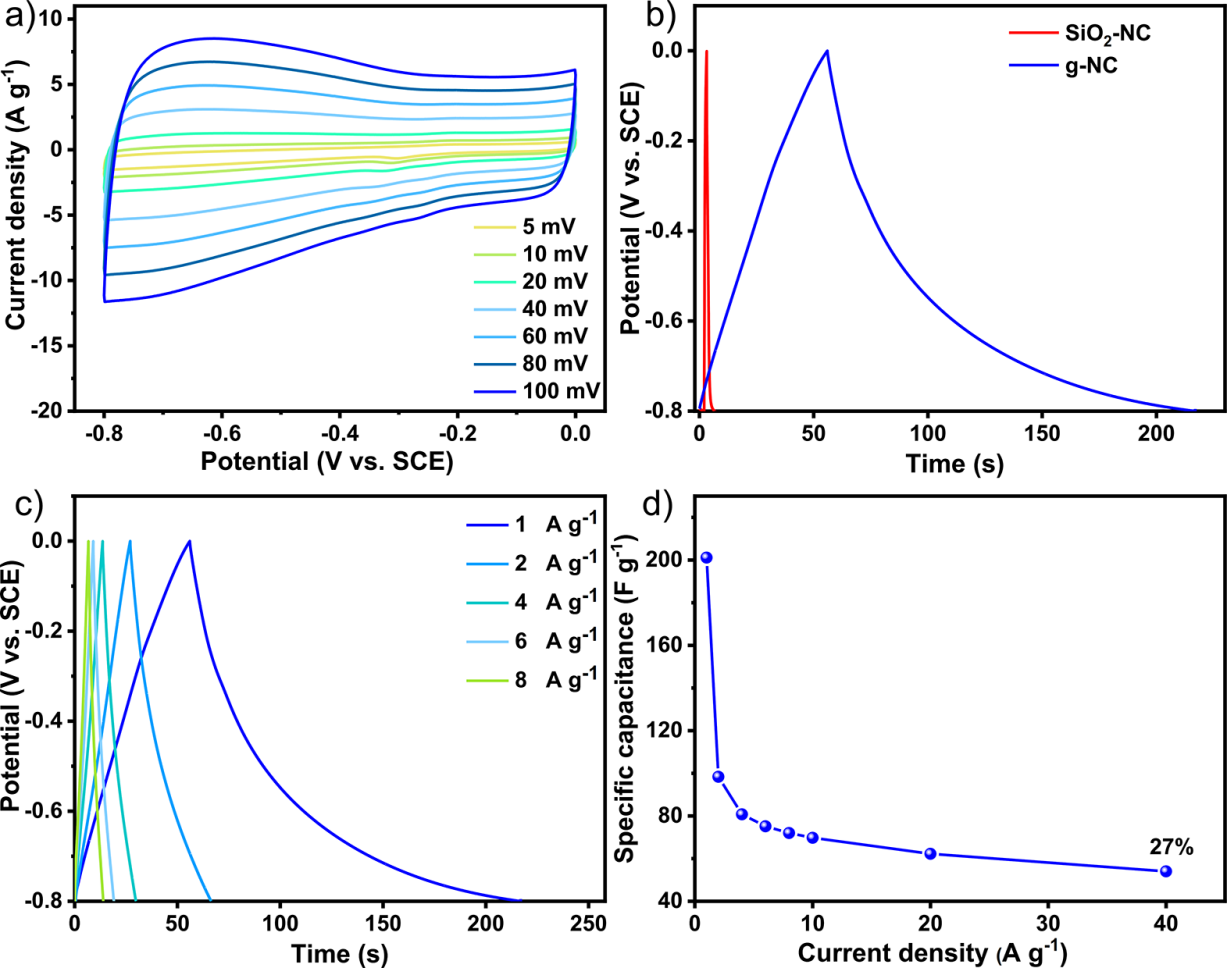
(a) CV curves of g-NC at different scan rates. (b) GCD curves of SiO_2_-NC and g-NC at 1 A g^–1^. (c) GCD curves of g-NC at current densities from 1–8 A g^–1^. (d) Specific capacitance at different current densities of g-NC.

The supercapacitance behavior of the g-NC was examined by CV and GCD methods. The CV voltammograms of the g-NC were taken in the potential range from 0 to −0.8 V (vs SCE) at various scan rates and represented in Figure 10a. We observed a linear increment of current at the electrode by increasing the scan rate. All voltammograms showed nearly an ideal rectangular shape indicating acceptable double-layer capacitive behavior and quick charging/discharging of the electrode. To understand the impact of silica etching, we also measured the SiO_2_–NC’s GCD curves at a current density of 1 A g^–1^ and compared them under identical conditions, depicted in Figure 10b. As can be seen, only a small specific capacitance was observed for SiO_2_–NC with less discharge time. However, in the case of g-NC, we observed a longer discharge time with significantly larger specific capacitance. This remarkable increase in the capacitance value can be ascribed to the activation of interlayers of g-NC blocked by silicate layers templating. Figure 10c shows the GCD curves of g-NC at different current densities from 1 to 8 A g^–1^. Their characteristic discharge curves showed distortion due to faradaic reactions, and, the specific capacitance (Figure 10d) decreased with the increase of current density. This decrease can be due to the fast reaction kinetics at high current density. The specific capacitance of g-NC against current density is shown in Figure 10d. As can be seen, the maximum value for g-NC was ca. 202 F g^–1^ at 1 A g^–1^, which is even more significant than its corresponding structure prepared with a similar pathway and reduced graphene oxide.^53,54^ Lastly, the stability of the g-NC in supercapacitance applications was also explored. After 10,000 cycles, g-NC retains 90% of its activity compared to a fresh sample, confirming its excellent stability. The results of ORR through electrocatalysis of g-NC are elucidated in the Supporting Information, and the relevant plots are illustrated in Figures S4 and S5. The *in situ* and *ex situ* Raman spectra of g-NC are also represented in Figures S6 and S7.

## 4 Conclusion

Layered silicate was utilized as a 2D hard template to produce a nitrogen-containing autonomously exfoliated porous graphitic carbon (g-NC). The chemical composition was found to be C_6.3_H_3.6_N_1.0_O_1.2,_ with graphitic nitrogen as the dominant configuration. Density functional calculations further indicated that graphitic nitrogen is the most stable configuration over the other two configurations, matching well with the XPS result. The order of nitrogen configuration stability was found as follows: graphitic > pyridinic > pyrrolic at the C to N ratio of ∼6:1. Further lattice dynamics calculations reveal that, in addition to increasing the surface area, pores substantially increase the defect in the graphitic carbon. The supercapacitance property of g-NC was higher than the conventionally prepared reduced graphene oxides. Notably, using an oxygen-rich layered material (here layered silicate) as a template for graphitic carbon synthesis besides the autogenous exfoliation and porosity generation may substitute oxygen species in carbon structure during the carbonization at high temperatures. In such cases, other layered materials, such as metal nitrides or sulfides, should be considered as templates.

## Supporting Information Available

Results and methodologies for electrochemical measurements and *in situ* Raman spectroscopy, results for the electrocatalytic oxygen reduction reaction, TG-DT profile for C_16_TMA-SiO_2_-mel, ATR-FTIR spectrum of melamine, CHNS analysis results of g-NC and SiO_2_–NC, output simulation data for the optimized g-NC structures (PDF)
Crystallographic data for graphitic, pyridinic, and pyrollic nitrogen configurations (ZIP)
