# Supplementary material for: Templated Synthesis of Exfoliated Porous Carbon with Dominant Graphitic Nitrogen
Source: ACS Mater Au. 2023 Feb 27;3(3):231–41. doi: 10.1021/acsmaterialsau.2c00074 (PMC10176611; doi:10.1021/acsmaterialsau.2c00074)
Supplement: Supplementary file 1 — mg2c00074_si_001.pdf [file mg2c00074_si_001.pdf]

Supporting Information

Templated-Synthesis of Exfoliated Porous Carbon with Dominant Graphitic Nitrogen

Esmail Doustkhah,[1,*] Ahmed Kotb,[2] Saeede Tafazoli,[1,3] Timuçin Balkan,[1,4,5]
Sarp Kaya,[1,3,5] Dorian A. H. Hanaor,[6] and M. Hussein N. Assadi[7]

[1]*Koç University Tüpraş Energy Center (KUTEM),* 34450 *Sarıyer, Istanbul, Turkey*
[2]*Chemistry Department, Faculty of Science, Al-Azhar University,* 71524 *Assiut, Egypt*
[3]*Materials Science and Engineering, Koç University,* 34450 *Sarıyer, Istanbul, Turkey*
[4]*n2STAR Koç University Nanofabrication and Nanocharacterization Center for Scientific and Technological Advanced Research,* 34450 *Sarıyer, Istanbul, Turkey*
[5]*Department of Chemistry, Koç University,* 34450 *Sarıyer, Istanbul, Turkey*
[6]*Fachgebiet Keramische Werkstoffe, Technische Universität Berlin,* 10623 *Berlin, Germany*
[7]*RIKEN Center for Emergent Matter Science,* 2–1 *Hirosawa, Wako, Saitama* 351–0198, *Japan*

(Dated: February 25, 2023)

I. OXYGEN REDUCTION REACTION MEASUREMENTS

A. Methods

1. *Electrochemical measurements*

The electrochemical performance of the prepared electrodes was investigated through a three-electrode system. A homogeneous ink was prepared as follows: first, as-prepared samples were ground, and then, 5.0 mg of the ground sample was dispersed into 950 µL of ethanol: water mixed solution (volume ratio of 1 : 3) and 50 µL of 5.0 wt% Nafion. After 60 min sonication, 5 µL of the suspension was dropped onto a glassy carbon electrode (GCE) with an area of 0.1256 cm^2 and dried slowly. The mass loading was 0.2 $mg\,cm^{-2}$. For GCE activation, the sample was pre-polished with 1 µm diamond, then 0.05 µm alumina powder, and washed with water. All electrochemical

* edoustkhahheragh@ku.edu.tr

measurements were carried out using CHI 842B instrument, the cyclic voltammetry (CV), linear sweep voltammetry (LSV) at 10 $mV\,s^{-1}$, and chronoamperometry ($i-t$) measurements were carried out in O_2 saturated 0.1 M KOH at ≈ 0.5 V *vs.* RHE. The electrolyte solution was purged with ultra-pure O_2 or N_2 for at least 1 h before starting the measurements. A platinum wire and silver/silver chloride (Ag/AgCl) were used as the counter and reference electrode, respectively. The potentials were expressed with regard to the reversible hydrogen potential electrode (RHE). The overall electron transfer numbers per oxygen molecule were calculated from the slope of the Koutecky-Levich plots according to the following equation:

$$\frac{1}{J} = \frac{1}{J_k} + \frac{1}{J_L} = \frac{1}{J_k} + \frac{1}{0.62nFC_0(D_0)^{2/3}\nu^{-1/6}} \times \frac{1}{\omega^{1/2}}, \qquad (Eq.\ SI)$$

where J is the current density, J_k is the kinetic current density, J_L is the diffusion-limited current density, n is the transferred electron number, F is Faraday constant ($F = 96485\ \mathrm{C\,mol^{-1}}$), C_0 is the concentration of O_2 ($1.2 \times 10^{-6}\ \mathrm{mol\,m^{-3}}$), D_0 is the diffusion coefficient of O_2 ($1.9 \times 10^{-5}\ \mathrm{cm^2\,s^{-1}}$), ν is the kinematic viscosity of the electrolyte ($0.01\ \mathrm{cm^2\,s^{-1}}$), and ω is the electrode rotating rate.

2. In situ Raman spectroscopy

Raman spectra were measured with a Renishaw inVia Raman system. A 50× long working distance (8 mm) objective was used. The wavelength of the excitation laser was 633 nm from a He−Ne laser. The laser power was 6 mW. Raman frequencies were calibrated using Si wafer spectra. All Raman spectra were measured over an acquisition time of 10 s and 1 accumulation. The Raman cell used for this measurement is shown in Figure S4. This cell configuration was employed to perform Raman spectroscopy at a biased electrode immersed in a very thin layer of electrolyte (0.1 M KOH). The drop-casted electrode was characterized at the following applied potentials (using chronoamperometry) in an O_2-saturated electrolyte.

B. Results

1. Electrocatalytic oxygen reduction reaction (ORR)

In addition to supercapacitance, we investigated the ORR activity for the oxygen reduction reaction in g−NC. Figure S4a represents LSV curves taken before and after silicate etching at 1600 rpm. For comparison, the LSV curve of commercial carbon black (Vulcan XC–72) measured as a reference commercial electrocatalyst was shown in the same Figure. Onset potential, half-wave

potential, and limiting current density at 0 V *vs.* RHE of these samples were extracted from LSV curves shown in Figure S4a and summarised in Figure S4b. Compared to the carbon black and SiO_2−NC, we obtained a better activity for g−NC in all three terms, indicating g−NC has a better catalytic activity for the ORR than the other samples. In detail, onset potential was enhanced from 0.72 V to 0.8 V *vs.* RHE, most probably due to the less or no activity of the silicate part. A similar trend was also observed for the half-wave potential. Moreover, limiting current density has also increased almost twice compared to SiO_2−NC. While SiO_2−NC shows nearly the same onset and half-wave potential as the carbon black (Vulcan XC–72), the limiting current value is much lower since the presence of SiO_2 in the structure raises the charge transfer resistance. Hence, the best limiting current value was reached after eliminating SiO_2, as expected for the g−NC sample. For electrocatalytic ORR, we have two already-accepted mechanisms in alkaline solutions: reduction via four electrons from O_2 to OH^- and the reduction of O_2 to H_2O^- via two electrons. To clarify the mechanism, we calculated the electron numbers using the Koutecky-Levich formula to find the relevant mechanism. Figure S5a exhibits LSV curves at various rotating speeds via a rotating disk electrode (RDE) and the Koutecky-Levich plots at multiple potentials for the g−NC in O_2 saturated 0.1 M KOH aqueous solution. As shown in Figure S5b, there was a linear relationship between the inverse of the square root of rotating speed ($\omega^{-1/2}$) and the inverse of measured current density (J^{-1}) irrespective of potential. The number of electrons in ORR was extracted from the slopes of each straight line and found to be between 2.4– 2.8 e, regardless of potential, indicating that a two-electron reduction reaction proceeded on these electrodes (Figure S5c). The stability measurement of the g−NC sample was also carried out under constant potential seen in Figure S5d. It was found that after 100 minutes, the initial current value decreased by 27%. By evaluating the outputs of the results, it can be claimed that g−NC can be an alternative candidate as a supporting carbon material for metal-based electrocatalysts instead of commercial carbon black. This way, it may be possible to produce more active electrocatalysts that show better onset and higher current due to the synergistic effect obtained between the metal and g−NC-based supporting material.

2. In situ Raman spectroscopy of g−NC in electrocatalysis

In the next step, we studied the stability of the material through *in situ* Raman spectroscopy in an electrochemical process using a cell running where the Raman spectra measurement is monitorable. The setup details are presented in Figure S3. We performed this experiment to

investigate any functional group alteration and stability of g−NC during the electrocatalysis. As shown in Figure S6, two prominent bands appeared in 1352 cm^{-1} and 1590 cm^{-1}, respectively, attributable to D and G bands. The intensity ratio of the D band (I_D) to the G band (I_G) intensity before starting the ORR reaction was 1.7. However, after applying voltage, a minor change occurs in the ID/IG ratio, representing that the material is stable during the electrocatalytic reaction while applying the voltage. Therefore, we did not see a remarkable change in the overall shape and the bands' intensities. A new shoulder at $\sim$ 1440 cm^{-1} appeared immediately after adding the electrolyte because of the electrolyte and g−NC interaction. This can be recognised by comparing the Raman spectra before and after adding the electrolyte, shown in Figure S7.

II. SUPPLEMENTARY FIGURES

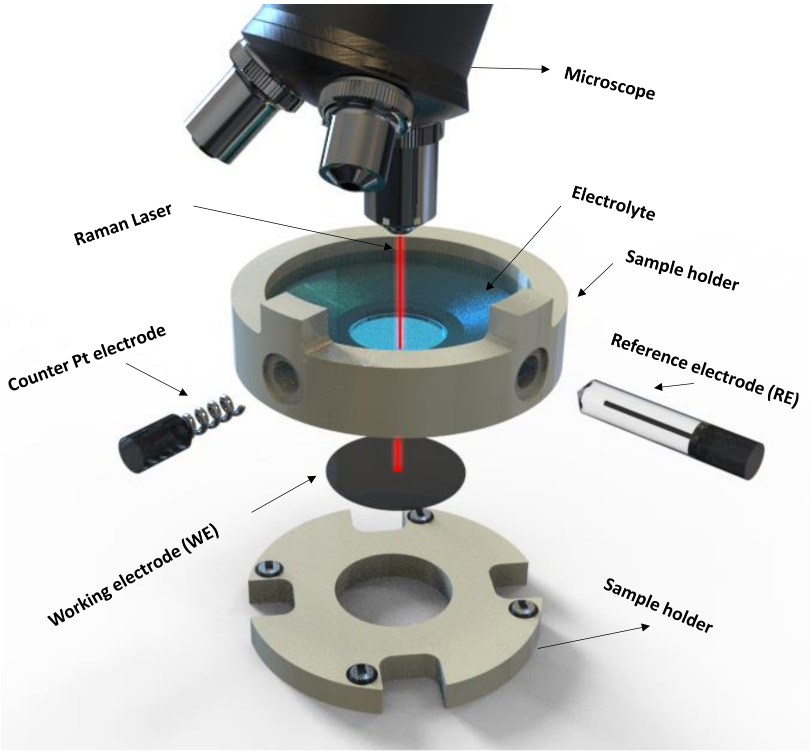

Figure S1. Schematic representation of the *in situ* Raman spectroscopy measurement during the oxygen reduction reaction.

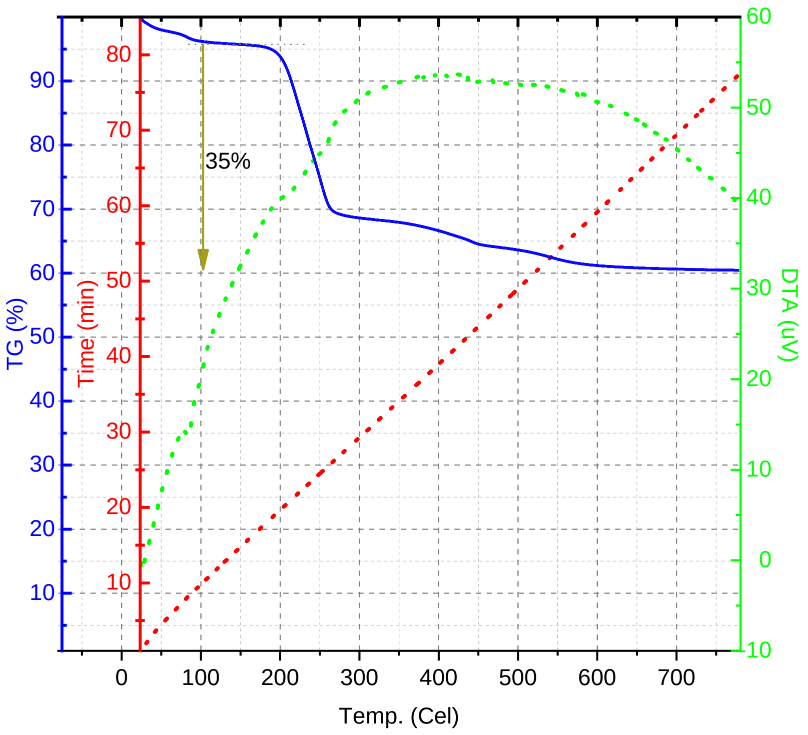

Figure S2. TG-DT profile of C_{16}TMA−SiO_2−mel run for 77 min.

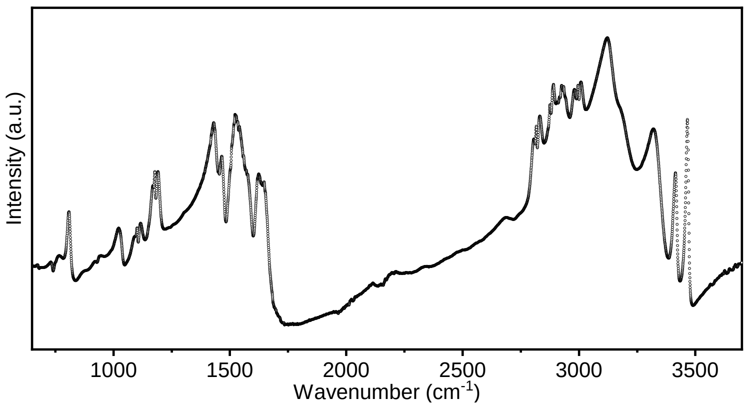

Figure S3. ATR-FTIR spectrum of melamine.

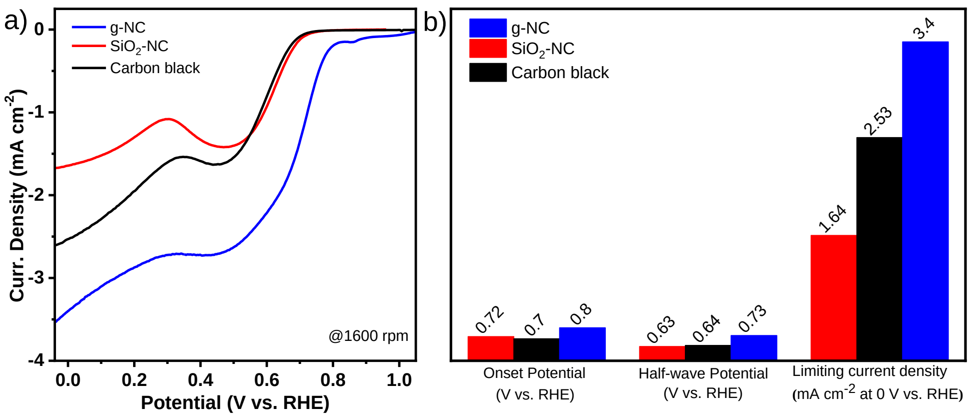

Figure S4. RDE voltammograms at 1600 rpm, b) Onset potentials, half-wave potentials and limiting current densities of g−NC, SiO_2−NC, and carbon black.

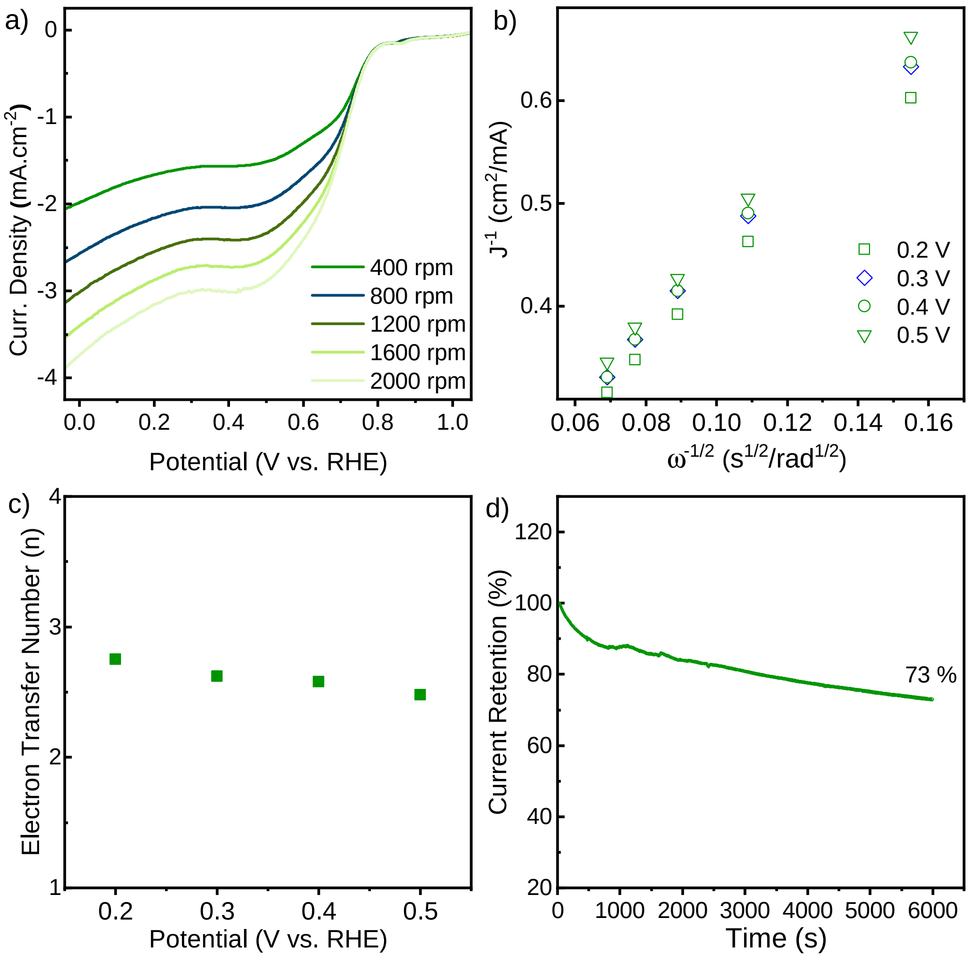

Figure S5. a) RDE voltammograms of g−NC in O_2 saturated 0.1 M KOH at different rotation rates. b) Koutecky-Levich plots at different potentials. c) Electron transfer numbers. d) Chronoamperometry results at 0.5 V *vs.* RHE.

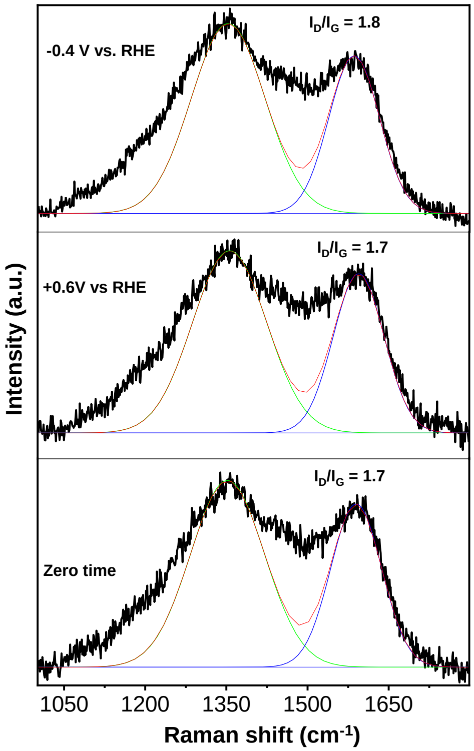

Figure S6. *In situ* Raman spectroscopy of g−NC in ORR reaction. Zero-time spectra represent the Raman spectra before applying any voltage to the electrolyte.

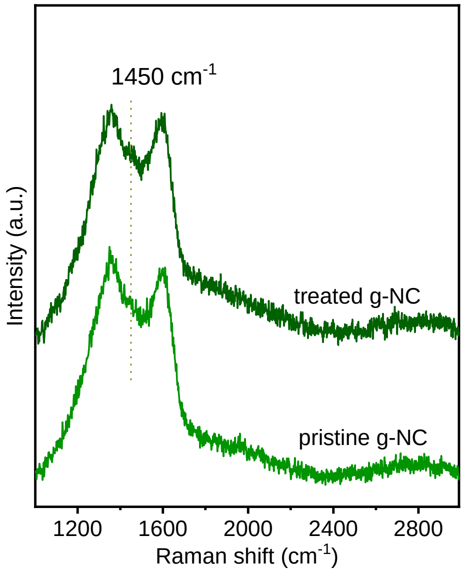

Figure S7. Raman spectroscopy of g−NC in dry and electrolyte-treated forms.

III. SUPPLEMENTARY TABLE

TABLE S1. CHNS analysis results of g−NC and SiO_2−NC materials.

Sample	Weight (mg)	%N	%C	%H
g−NC	3.408	12.44	67.27	3.17
SiO_2−NC	2.282	4.45	8.20	1.44

IV. OPTIMISED STRUCTURES

```
Graphitic-C6N1
   1.00000000000000
     8.7971709687323791    0.0007680889621788    0.0000000000000000
    -7.7822328726382048    4.1020810764994780    0.0000000000000000
     0.0000000000000000    0.0000000000000000    6.2402932958211244
   C    N
    24     4
Direct
  0.8232269944017503  0.8232269944017503  0.5000000000000000
  0.1583815315864143  0.1583815315864143  0.5000000000000000
  0.2544915055032604  0.3978814366102554  0.5000000000000000
  0.3978814366102554  0.2544915055032604  0.5000000000000000
  0.9731876033808220  0.6864947507240160  0.5000000000000000
  0.6864947507240160  0.9731876033808220  0.5000000000000000
  0.1120002465851186  0.5388298214695979  0.5000000000000000
  0.5388298214695979  0.1120002465851186  0.5000000000000000
  0.5882315703810193  0.7326188562639047  0.5000000000000000
  0.7326188562639047  0.5882315703810193  0.5000000000000000
  0.8747726150892134  0.4455489085625643  0.5000000000000000
  0.4455489085625643  0.8747726150892134  0.5000000000000000
  0.1611941533838177  0.1611941533838177  0.0000000000000000
  0.8260519550489960  0.8260519550489960  0.0000000000000000
  0.5910370912583929  0.7354240218045689  0.0000000000000000
  0.7354240218045689  0.5910370912583929  0.0000000000000000
  0.4483391182678531  0.8775600667297994  0.0000000000000000
  0.8775600667297994  0.4483391182678531  0.0000000000000000
  0.2573081222641989  0.4006977954007382  0.0000000000000000
  0.4006977954007382  0.2573081222641989  0.0000000000000000
  0.9759862055951771  0.6892946726313198  0.0000000000000000
  0.6892946726313198  0.9759862055951771  0.0000000000000000
  0.1148048348744553  0.5416320827721250  0.0000000000000000
  0.5416320827721250  0.1148048348744553  0.0000000000000000
  0.0171611461847547  0.3026208106813684  0.5000000000000000
  0.3026208106813684  0.0171611461847547  0.5000000000000000
  0.3054102996211796  0.0199517909233151  0.0000000000000000
  0.0199517909233151  0.3054102996211796  0.0000000000000000
```

Data S1: The optimised structure of C_6N_1 with graphitic nitrogen in POSCAR format.

```
Pyridinic-C6N1H3
   1.00000000000000
     4.9943352087569917   -0.0618180861984125    0.0000000000000000
     2.4698246621835342    4.3413329510128813    0.0000000000000000
     0.0000000000000000    0.0000000000000000    6.5303958895551277
   H    C    N
     6    12     2
Direct
  0.1397852817219203  0.3972259814528343  0.4694615117345791
  0.8602147182780797  0.6027740185471657  0.9694615117345791
  0.6027740185471586  0.8602147182780797  0.9694615117345720
  0.3972259814528414  0.1397852817219203  0.4694615117345791
  0.3824187121016038  0.3824187121016038  0.2078121535256301
  0.6175812578984008  0.6175812578984008  0.7078121535256301
  0.7991346245115878  0.3106931707675002  0.3339608451224834
  0.2008653754884051  0.6893067992324973  0.8339608451224834
  0.6893067992324973  0.2008653754884051  0.8339608451224834
  0.3106931707675002  0.7991346245115878  0.3339608451224834
  0.0613979693245952  0.5160454116454858  0.8792731454954676
  0.9386020306754048  0.4839545883545142  0.3792731454954676
  0.4839545883545213  0.9386020306754048  0.3792731454954676
  0.5160454116454787  0.0613979693245952  0.8792731454954676
  0.9677375937289625  0.9677375707289571  0.3413125945087359
  0.0322624062710304  0.0322624062710375  0.8413125645087334
  0.8041364649542899  0.8041364649542828  0.3520415173452136
  0.1958635350457172  0.1958635050457289  0.8520414873452182
  0.4882637385925506  0.4882637385925506  0.2780426519153707
  0.5117362614074494  0.5117362614074494  0.7780426819153732
```

Data S2: The optimised structure of $C_6N_1H_3$ with pyridinic nitrogen in POSCAR format.

```
Pyrrolic-C7N1H3
   1.00000000000000
     7.9366705900643444    0.0000000000000000   -0.3907652171226898
     0.0000000000000000    6.2185429877456899    0.0000000000000000
    -4.3068136185613843    0.0000000000000000    6.9022303924303348
   H    C    N
    12    28     4
Direct
  0.5597452519513340  0.5000000000000000  0.4192084200995723
  0.7061440848186962  0.5000000000000000  0.7962467256837300
  0.7057114208964137  0.5000000000000000  0.5974891048044029
  0.1413278408297813  0.5000000000000000  0.8466792075024614
  0.4118761129206021  0.5000000000000000  0.9593061608381177
  0.2400526141901977  0.5000000000000000  0.7911075748095584
  0.7633462078071318  0.0000000000000000  0.2418155844681849
  0.2270156070019098  0.0000000000000000  0.4053216124415044
  0.9448419924476283  0.0000000000000000  0.4164276074561357
  0.5936121648810868  0.0000000000000000  0.2092840125354272
  0.0495644241067978  0.0000000000000000  0.2422226600456057
  0.2957186481522029  0.0000000000000000  0.6194465461729592
  0.9591510269578691  0.5000000000000000  0.1327622718866479
  0.0971950861179565  0.5000000000000000  0.3427892717498366
  0.0433351521780452  0.5000000000000000  0.4805296712676537
  0.0619612843608692  0.5000000000000000  0.0231168330618061
  0.2991267678733891  0.5000000000000000  0.1519409135193186
  0.4024108846287504  0.5000000000000000  0.3558561069971162
  0.3166502577937322  0.5000000000000000  0.4594223672860807
  0.4015644360631327  0.5000000000000000  0.6708552870190658
  0.7407591760780221  0.5000000000000000  0.0628305616808049
  0.9977814272216321  0.5000000000000000  0.8203521849361195
  0.8467173555395888  0.5000000000000000  0.4409605111546568
  0.6759923451283854  0.5000000000000000  0.2167638802129730
  0.4559554920201307  0.5000000000000000  0.1092487570121037
  0.6054900476498304  0.5000000000000000  0.8454948522083399
  0.0038950188426696  0.0000000000000000  0.8648942861148825
  0.8990268423326313  0.0000000000000000  0.6544300572877830
  0.0106536360499021  0.0000000000000000  0.5727145090873620
  0.8538263349360165  0.0000000000000000  0.9150900019754857
  0.6245347947056956  0.0000000000000000  0.7891958237291234
  0.5414679493215147  0.0000000000000000  0.5780437386797530
  0.6811634385434004  0.0000000000000000  0.5240400229559370
  0.5620295413761482  0.0000000000000000  0.3161799091665429
  0.2306701306676899  0.0000000000000000  0.0003352784701036
  0.9060361261461338  0.0000000000000000  0.1051966521663630
  0.2168504382252081  0.0000000000000000  0.6901224486372044
  0.3453350899006224  0.0000000000000000  0.8977725101417988
  0.5573907206498205  0.0000000000000000  0.9278248646419698
  0.3558751199892214  0.0000000000000000  0.2270566016990898
  0.2254819011570959  0.5000000000000000  0.6629400362800482
  0.8440878573041104  0.5000000000000000  0.6196606699304752
  0.7416874226065886  0.0000000000000000  0.1080480918506694
  0.3482506336304212  0.0000000000000000  0.3978458713351998
```

Data S3: The optimised structure of $C_7N_1H_3$ with pyrollic nitrogen in POSCAR format.
